# Impact of Cancer Awareness Drive on Generating Awareness of and Improving Screening for Cervical Cancer: A Study Among Schoolteachers in India

**DOI:** 10.1200/JGO.17.00074

**Published:** 2018-02-22

**Authors:** Abhishek Shankar, Shubham Roy, Goura Kishor Rath, Abhijit Chakraborty, Vineet Kumar Kamal, Aalekhya Sharma Biswas

**Affiliations:** **Abhishek Shankar** and **Goura Kishor Rath**, Dr BR Ambedkar Institute Rotary Cancer Hospital; **Abhishek Shankar**, **Goura Kishor Rath**, and **Vineet Kumar Kamal**, All India Institute of Medical Sciences; **Shubham Roy**, Vardhman Mahavir Medical College and Safdarjung Hospital, New Delhi; **Abhijit Chakraborty**, Netaji Subhas Chandra Bose Cancer Research Institute; and **Aalekhya Sharma Biswas**, Maulana Abul Kalam Azad University of Technology, Kolkata, India.

## Abstract

**Purpose:**

Cervical cancer is the second most common cancer in India. Our study assessed the level and impact of awareness programs in the adoption of safe practices in prevention and early detection.

**Methods:**

This assessment was part of a Pink Chain Campaign, the mission of which is to fight cancer. During cancer awareness events from 2013 to 2015 at various women’s colleges in different parts in India, a pretest related to cervical cancer was followed by an awareness program. A post-test was conducted 6 months and 1 year later.

**Results:**

A total of 872 of 985 teachers participated in the study, for a response rate of 88.5%. Mean age of the population was 42.4 years. There was a significant increase in the level of knowledge regarding cervical cancer at 6 months, which was sustained at 1 year. Regarding cervical cancer screening, knowledge and practice of the Papanicolaou (Pap) test as a screening test for cervical cancer among teachers were changed significantly at 6 months and 1 year. More than 75% of teachers were educated by physicians about the Pap test. At the time of the post-test, there was a significant change in alcohol and smoking habits. The main reasons for not undergoing a screening test were ignorance (50%), lethargic attitude (44.8%), and lack of time (34.6%).

**Conclusion:**

The level of knowledge of cervical cancer was poor. A significant increase in the level of knowledge of cervical cancer among the population was found after this study. To inculcate safe lifestyle practices, awareness programs should be conducted more widely and frequently.

## INTRODUCTION

Cancer is a major public health problem both in our country and worldwide because of its disease burden, fatality, and tendency toward increased incidence.^1^ Globally, cervical cancer is the second most prevalent cancer among all populations and third most common type of cancer after breast and lung cancers among women.^2^ The global burden of cervical cancer is disproportionately high in developing countries, where 85% of the estimated 493,000 new cases and 273,000 deaths occur worldwide.^3^ India, which accounts for one sixth of the world’s population, also bears one fifth of the world’s cervical cancer burden.^4^ Over the past 40 years, mortality resulting from carcinoma of the cervix has fallen because of improved treatment and the introduction of national screening programs.^5^ The Papanicolaou (Pap) test is the main screening method used for the secondary prevention of cervical cancer. It can detect precancerous cells easily.^6^ The Pap test is an effective method of detecting, preventing, and delaying the progress of cervical cancer.^2,7^

Cervical cancer is the second most common cause of cancer mortality among women in India; however, it is a largely preventable disease.^8^ In India, the cervical cancer incidence is one fifth of the world’s incidence.^9^ Awareness and health-seeking practices have been shown to be poor in many developing countries, necessitating the need for proper awareness programs.^10-12^ With improvement in cancer technology, we have been able to improve quality of life, but improvement in survival is still questionable. In India, late presentation is attributed to many factors, notably a lack of knowledge and awareness of and a lethargic attitude toward safe health practices.

Several studies have shown that the knowledge of cervical cancer and practices for early detection are at a low level among women.^12^ Because early detection is one way to reduce morbidity and mortality resulting from cervical cancer, there is work going on, although limited, to increase knowledge, safe practices, and attitudes regarding cervical cancer among schoolteachers in India. The purpose of this study is to measure the level of awareness of cervical cancer risk factors and safe practices among college teachers of different states of India and the impact of awareness programs on changes in the adoption of safe practices for prevention and early detection.

## METHODS

This assessment was part of the Pink Chain Campaign, the mission of which is to fight against and curb the spread of cancer; the foundation is dedicated to creating awareness throughout India of self-care for the protection of self-worth and maintenance of healthy, disease-free lifestyles. For this study, our target population for spreading basic knowledge and awareness was schoolteachers, because we hope after the study they can increase the basic awareness level of the larger society. During the cancer awareness events from 2013 to 2015 at women’s colleges in different states in India (ie, Delhi, Mumbai, and Jaipur), a pretest of knowledge, attitudes, and practices related to cervical cancer was conducted by questionnaire. The questionnaire was formulated on the basis of our observations of such knowledge, attitudes, and practices and validated in our pilot study of 186 college teachers in various states of India.

Official permission was granted to conduct the pretest and post-test and use databases at 6 months and 1 year from college authorities before the awareness campaign. All teachers received an explanation of the study, and after that, pretest and post-test questionnaires were given to those teachers who volunteered to take part. College teachers included in this study were teaching arts, science, and commerce to graduate students in Delhi, Mumbai, and Jaipur in India. The pretest was followed by an awareness program consisting of lectures by oncologists working at medical institutions on preventive aspects of cervical cancer, with an interactive session. A post-test using the same questionnaire was conducted at the end of the interactive session. Personal details of all teachers who participated in the awareness program were collected. The Pink Chain Campaign is a nationwide campaign that started with promoting awareness of breast cancer. Over a period of time, the Pink Chain Campaign also started promoting awareness of other common cancers (eg, cervical, lung, and head and neck cancers) from the same platform. Literature related to cancer awareness was sent regularly (every 3 months) for 1 year to e-mail addresses provided. After 6 months and 1 year, the same questionnaires were mailed to the participant e-mail addresses provided at the time of the start of the program to determine any changes in practice.

Data are presented as frequency (%) using descriptive analysis. To compare the pretest with post-test changes for knowledge about various aspects of carcinoma of the cervix, a McNemar test was applied at the bivariable level. All *P* values < .05 were considered significant. All statistical analyses were performed using STATA software (version 12.1; STATA, College Station, TX).

## RESULTS

**Fig 1:**
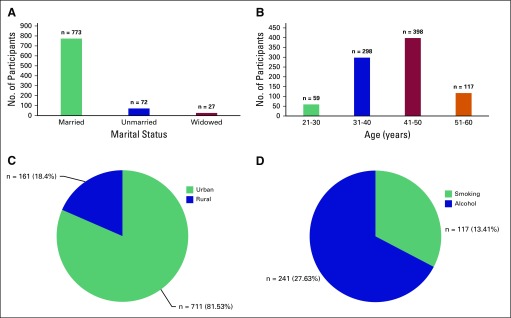
Sociodemographic variables of studied population: (A) marital status, (B) age, (C) demography, and (D) alcohol and smoking habits.

A total of 872 of 985 teachers participated in the study (overall response rate, 88.5%); 113 teacher assessment forms were either not filled in or incomplete. In the information provided, the e-mail addresses of 787 teachers were available. After 6 months and 1 year, 612 and 504 teachers responded to the same questionnaires, respectively. The mean age of the study population was 41.6 years (standard deviation, 8.3 years). Sociodemographic details of the study population are shown in Figure 1. The age group of 31 to 50 years included 696 teachers (79.81%). Most of the teachers (81.53%) were from urban backgrounds. Among the teachers who were asked about smoking habits and alcohol consumption, 117 (13.41%) were smokers, and 241 (27.63%) were alcoholics. Most of the teachers were married (88.6%; n = 773). The teachers who were nonsmokers and avoided alcohol consumption maintained those habits, at least over the study period. Among those who smoked and consumed alcohol before the campaign, changes in smoking and alcohol consumption were noted at 6 months and 1 year. A decrease in frequency was seen in 11% for smoking and 16% for alcohol at 1 year. Approximately 21% and 43% of teachers had quit smoking and consuming alcohol, respectively, at the end of 1 year. Six months and 1 year after the awareness campaign, there was a significant change in alcohol and smoking habits.

Knowledge regarding various aspects of carcinoma of the cervix is summarized in Table 1. The most common risk factors indicated by teachers were family history of cervical cancer (47.24%), smoking (33.1%), and genital hygiene (32.4%). Surprisingly, it was found that most teachers (n = 487; 55.84%) were unaware of the symptoms of cervical cancer before the awareness program. After the test, their knowledge significantly increased. Table 2 summarizes the changes in knowledge regarding the Pap test before and after the awareness and screening campaign. Knowledge and practice of the Pap test as a screening test for cervical cancer among teachers were changed significantly (*P* ≤ .001) at 6 months and 1 year. Table 3 lists the sources of knowledge regarding the Pap smear among the studied population. It was observed that most teachers (75%) were educated by physicians regarding the Pap test. Table 4 represents the reasons for not undergoing a screening test. The major reasons were ignorance (83.5%), lethargic attitude (29.3%), and lack of time (10.9%).

**Table 1:**
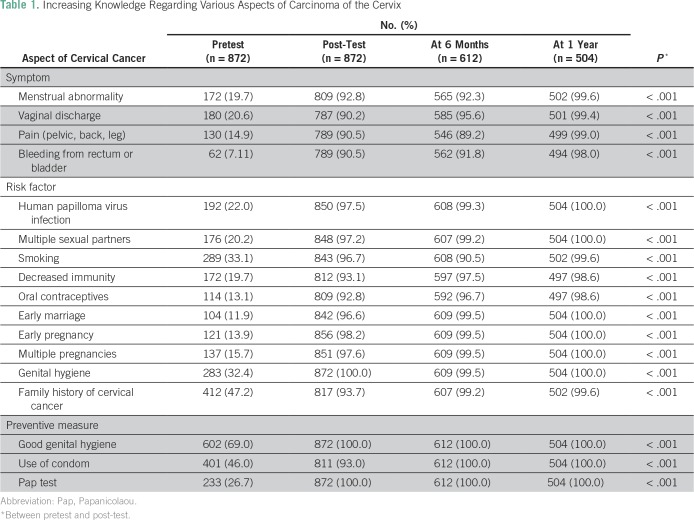
Increasing Knowledge Regarding Various Aspects of Carcinoma of the Cervix

**Table 2:**
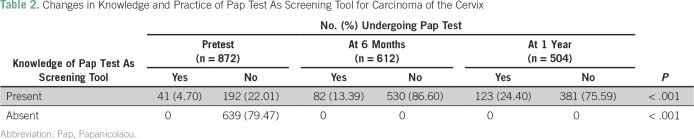
Changes in Knowledge and Practice of Pap Test As Screening Tool for Carcinoma of the Cervix

**Table 3:**
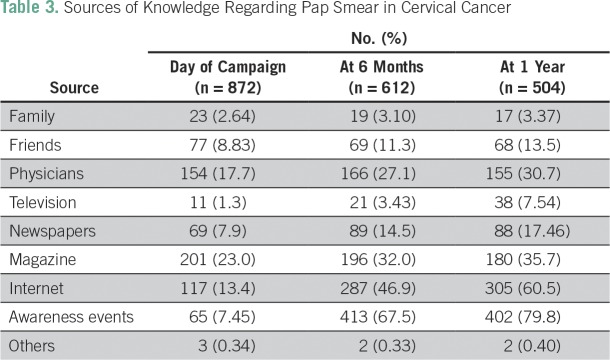
Sources of Knowledge Regarding Pap Smear in Cervical Cancer

**Table 4:**
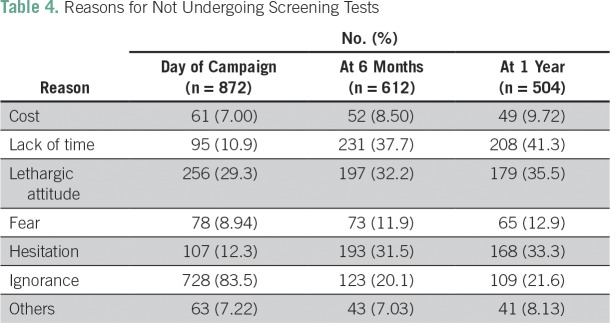
Reasons for Not Undergoing Screening Tests

## DISCUSSION

A prerequisite for early diagnosis is to have knowledge of the symptoms of cancer so a physician is consulted as early as possible.^13^ We found that most of the women were unaware of the risk factors for cancer of the cervix. Similar findings were noted in another study.^14^ The study by Basu et al^15^ in 2014 found cervical cancer was known to only 38%. A study from North Bengal, India, by Raychoudhuri and Mandal^16^ showed that 87% of women were aware of the term cancer, but few knew about cervical cancer. It was observed that 96.4% of the population was unaware of the causes of the disease, 93.7% unaware of the signs or symptoms, and 96.4% unaware of ways to prevent cervical cancer. A study by Siddharthar et al^17^ showed that less than half of the study population was aware of cervical cancer, and only one third of women had knowledge about its risk factors and symptoms.

Smoking is an important risk factor in developing cervical cancer. Smokers have human papillomavirus cervical infections much longer and are less likely to clear them than nonsmokers. Cigarette smoking (10.3%), long-term use of the oral contraceptive pill (16.3%), poor genital hygiene (7.5%), and a sexual partner with multiple partners (15%) were considered the major risk factors for cervical cancer. Of the symptoms of cervical cancer, bleeding during or after sex (15.3%), vaginal bleeding after menopause (10.3%), pain during sex (10%), and persistent low back pain (9.8%) were considered more frequently by the women in the study. Only 7.3% of the women considered themselves at risk for cervical cancer.

Regarding preventive measures, the Pap test was known to only 35% of teachers in this study. In the study by Raychoudhuri and Mandal,^16^ 90.5% of participants were unaware of the Pap test as a screening procedure, although 84.6% were willing to undergo the test, because they felt it would benefit them in the long run. This is in agreement with a study conducted in Kolkata.^18^

Sources of knowledge about screening modalities for cervical cancer were magazines, newspapers, and the Internet in a majority of cases, but physicians were important source for the Pap smear. According to the women surveyed, the main barrier for using early diagnostic services for cervical cancer was the lack of awareness about symptoms and of the availability of tests. Similar findings were mentioned by other studies.^14^ Lack of awareness lowers the possibility of early diagnosis despite availability of a screening test. Nearly half of the women had a favorable attitude toward or were willing to use early detection methods if they were available. Hence, lack of awareness was the main barrier to early detection.

Our findings suggest that awareness through mass media may not be sufficient in changing attitudes or practices. Other barriers were lethargic attitude (44.87%), economic barriers (time and money), availability, fear, and hesitation. Nessa et al^19^ showed that more emphasis must be placed on use of print and audiovisual media in cervical cancer prevention. Tan et al^20^ in 2010 showed that mass media and education were the most common sources of information on cervical cancer. The section of the population aware of cervical cancer came to know about this disease through friends or media like radio and television in a study in North Bengal, India.^16^ Cervical cancer education continues to pose a challenge to the health care system in developing countries and countries with limited resources. Community-based cancer awareness and education, which can help to improve the minimization of health disparities, are frequently needed at many levels.^21^

In summary, the risk factors and symptoms of cervical cancer were generally not well known. The level of knowledge of breast cancer risk factors, symptoms, and screening methods was high as compared with those of cervical cancer. There was a significant increase in the level of knowledge regarding risk factors, symptoms, and screening tests for cervical cancer at 6 months, and this was sustained at 1 year. To inculcate safe practices in lifestyles, awareness programs such as the Pink Chain Campaign should be conducted more widely and frequently, and knowledge should also be attained through physicians, who are the first point of contact with the health system. Therefore, creating awareness among health care providers is another issue to be considered. It is important to create awareness through educational programs on cancer prevention, preventable cancer risk factors, benefits of early diagnosis, and availability of screening facilities.

In developing countries such as India, cancer increases the burden of health care. With the advancement of civilization and modern lifestyles, cancer incidence is increasing day by day. One of the main causes in unawareness. Through our Pink Chain Campaign awareness programs, we are committed to conducting knowledge and awareness camps and sometimes free health checkups by experienced physicians. Through camps, we came to realize that a majority of cancer cases are lifestyle related. This can only be prevented by proper awareness camps. Therefore, cancer detection and awareness camps are essential and important to reducing the huge burden of cancer. We are creating awareness among the community through educational programs on different types of cancer prevention. One of our studies on awareness of breast cancer was published in 2017.^22^ In 2015, we published articles on breast and cervical cancers,^12^ in which we reported a study of cancer awareness events from 2011 to 2012 involving 156 teachers. In the current article, we discuss the impact of a cervical cancer awareness program that we conducted from 2013 to 2015. This is an extension of our published study, now including more participants (newly included). The results are based on data from 872 individuals who were covered during the time periods.

In conclusion, we are on the verge of a sea change in cervical cancer prevention. However, without a commitment to, and concomitant investment in, understanding and overcoming the psychosocial, institutional, and access barriers that perpetuate health disparities, these revolutionary technologic developments will be for naught. There was a significant increase in the level of knowledge regarding risk factors, symptoms, and screening tests for breast cancer at 6 months, and this was sustained at 1 year. It is important to create awareness among the community through educational programs on cancer prevention, preventable cancer risk factors, benefits of early diagnosis, and availability of screening facilities.

The use of this study positively influenced knowledge of cervical cancer risk factors, symptoms, and types of screening and increased screening rates in screening-naïve women. Given the high acceptance of respondents, it is premature to abandon this approach completely as a type of cancer education until it is tested again and paired with the practical information that is needed to schedule screening. Future interventions should also focus on methods to increase the role of health care providers in screenings.
